# Volume replacement with diced acellular dermal matrix in oncoplastic breast-conserving surgery: a prospective single-center experience

**DOI:** 10.1186/s12957-020-01835-6

**Published:** 2020-03-24

**Authors:** Hongki Gwak, Ye-Won Jeon, Seung-Taek Lim, Seon-Young Park, Young-Jin Suh

**Affiliations:** Division of Breast and Thyroid Surgical Oncology, Department of Surgery, St. Vincent’s Hospital, College of Medicine, The Catholic University of Korea, 93 Jungbu-daero, Paldal-gu, Seoul, Suwon 16247 Republic of Korea

**Keywords:** Acellular dermis, Breast-conserving surgery, Breast cancer, Breast reconstruction

## Abstract

**Background:**

Several studies have reported the use of acellular dermal matrix in breast reconstruction. However, the primary role of acellular dermal matrix in these studies was to support the implant; there are no reports on the use of acellular dermal matrix exclusively as volume replacement. Thus, we aimed to evaluate the safety and effectiveness of filling of the defect with acellular dermal matrix in oncoplastic breast-conserving surgery.

**Methods:**

We prospectively recruited 120 adult breast cancer patients who were scheduled to undergo oncoplastic breast-conserving surgery with acellular dermal matrix filling from 2017 to 2018. Intraoperatively, diced human acellular dermal matrix measuring 3–5 mm was used on each side to fill in the excisional defect immediately. After 6 months, satisfaction of the patients and surgeons with overall and cosmetic outcomes was evaluated with a survey using a 10-point scale. Postoperative complications were assessed at 2 weeks and 6 months postoperatively.

**Results:**

Of the 117 patients who were evaluated for their satisfaction, 94.0% were strongly satisfied with the cosmetic outcomes and 90.4% were strongly satisfied overall. Patient overall satisfaction scores were higher than surgeon satisfaction scores (*p* < 0.001). Of the 117 patients who underwent evaluation of complications 6 months postoperatively, six (5.1%) had hematoma and seven (6.0%) had seroma. The overall reoperation rate due to complications was 8.5%. Only two patients needed acellular dermal matrix removal due to hematoma and inflammation.

**Conclusion:**

Oncoplastic breast-conserving surgery with acellular dermal matrix filling of defects can be performed safely with high cosmetic satisfaction.

**Trial registration:**

ICTRP, KCT0003886; retrospectively registered May 3, 2019, http://apps.who.int/trialsearch/Trial2.aspx?TrialID=KCT0003886

## Background

Oncoplastic breast-conserving surgery (BCS) has been developed to overcome the cosmetic disadvantages of conventional BCS and has become a commonly used procedure in patients with breast cancer [1]. Oncoplastic BCS can be divided into volume displacement and volume replacement techniques [2]. In Asian women, the size of the breast is relatively small, whereas the size of the tumor is large [3–5]. Therefore, volume displacement with the patient’s own breast tissue only is difficult to achieve cosmetically satisfying results. In small to medium-sized breasts, the replacement technique has shown better results [6].

Acellular dermal matrix (ADM) contains collagen, elastin, proteoglycans, laminin, and a basement membrane. These materials serve as pillars for reepithelialization, neovascularization, and fibroblast infiltration [7]. ADM does not evoke an immune response and is widely used in burn care and reconstructive surgery [7–9]. In breast surgery, ADM is used in more than 75% of immediate tissue expander reconstruction procedures to support the implant [10]. However, the role of ADM in these procedures is only supportive, and no method has been described as filling the defects during BCS exclusively with ADM. Before starting this study, we performed BCS using ADM in 15 cases. None of the patients had immediate postoperative surgical site infections or red breast syndrome (RBS). In these cases, we rolled the ADM plate into a cylindrical shape or cut it into a crepe cake. Since each surgeon designed the ADM for each operation, the results were different according to the operator’s scissoring skills. In addition, because it is difficult to reproduce the shape of the breast, which changes according to a woman’s posture, some patients complained that lumps were detectable through palpation in certain positions. Therefore, we found that filling the breast defect using small cubes was ideal. We, therefore, performed oncoplastic BCS using immediate diced ADM filling, and aimed to evaluate its safety and the levels of patient satisfaction.

## Methods

### Patients

**Fig. 1:**
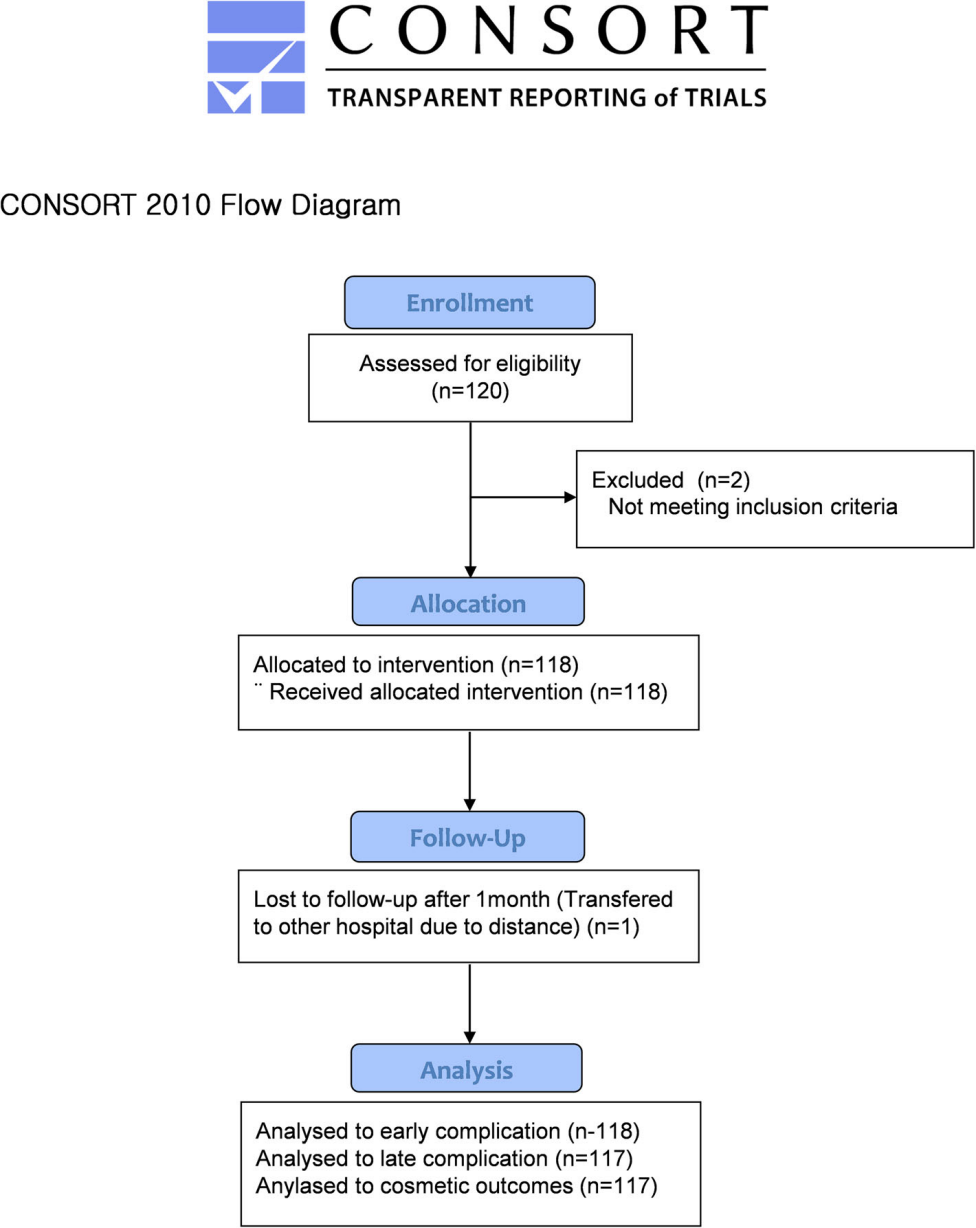
Flow diagram of participants

This prospective study consecutively enrolled 120 patients aged 20–80 years with breast cancer who desired BCS between December 2017 and August 2018. All patients agreed to the volume replacement procedure with ADM. We included patients regardless of the size and location of the mass in the breast. Patients with bilateral and multicentric breast cancer were excluded. Further exclusion criteria were infectious diseases, autoimmune diseases, blood clotting disorders, and inflammatory infections before the operation (Fig. 1).

We recorded demographic data, medical history, tumor stage, type and location, resection volume, and the administration of neoadjuvant chemotherapy for all patients.

All participants in this study provided written informed consent for the storage of their medical information in the database and the use of this information for research purposes. The study protocol was approved by the Institutional Review Board of the Catholic University of Korea (VC17OESI0168). All procedures were performed in accordance with the ethical standards of the institutional and/or national research committee and with the 1964 Helsinki Declaration and its later amendments.

### Procedure

**Fig. 2:**
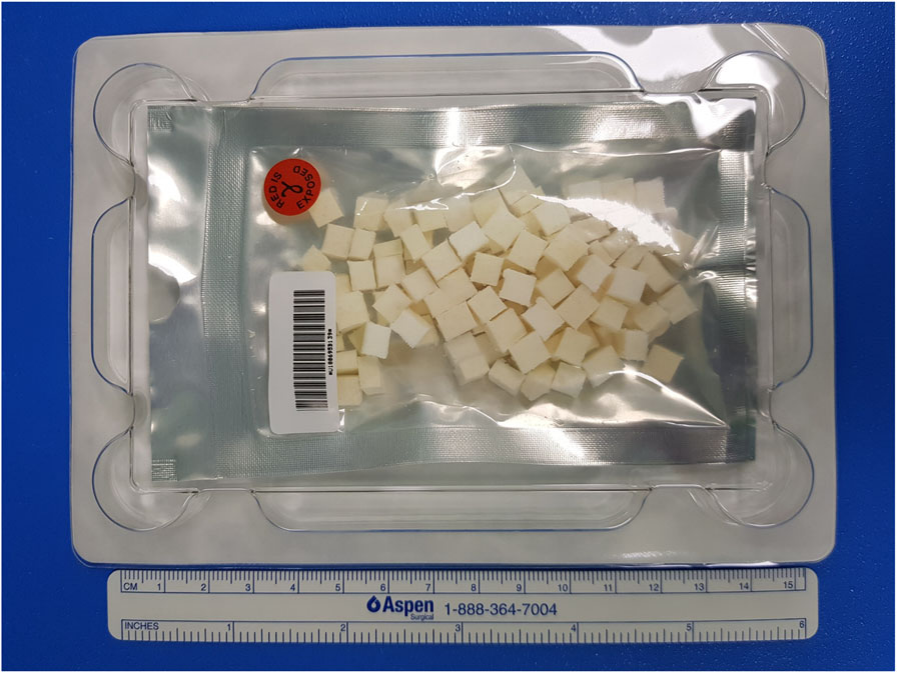
Diced acellular dermal matrix

In this study, the crosslinked human ADM (Megaderm®, L&C Bio, Seoul, Korea) derived from donated human skin in USA tissue banks following the guidelines of the American Association of Tissue Banks and the US Food and Drug Administration was used [6]. After removal of the epidermal and dermal cells from the fresh human cadaver skin, electron beam irradiation is applied to the remaining acellular dermal layer to remove viruses, bacteria, and spores. The product is a 5 × 6 cm square plate with a thickness of 3–5 mm. Each ADM square was diced and packed by the manufacturer and was immersed in normal saline for 30 min in the operating room (Fig. 2).

**Fig. 3:**
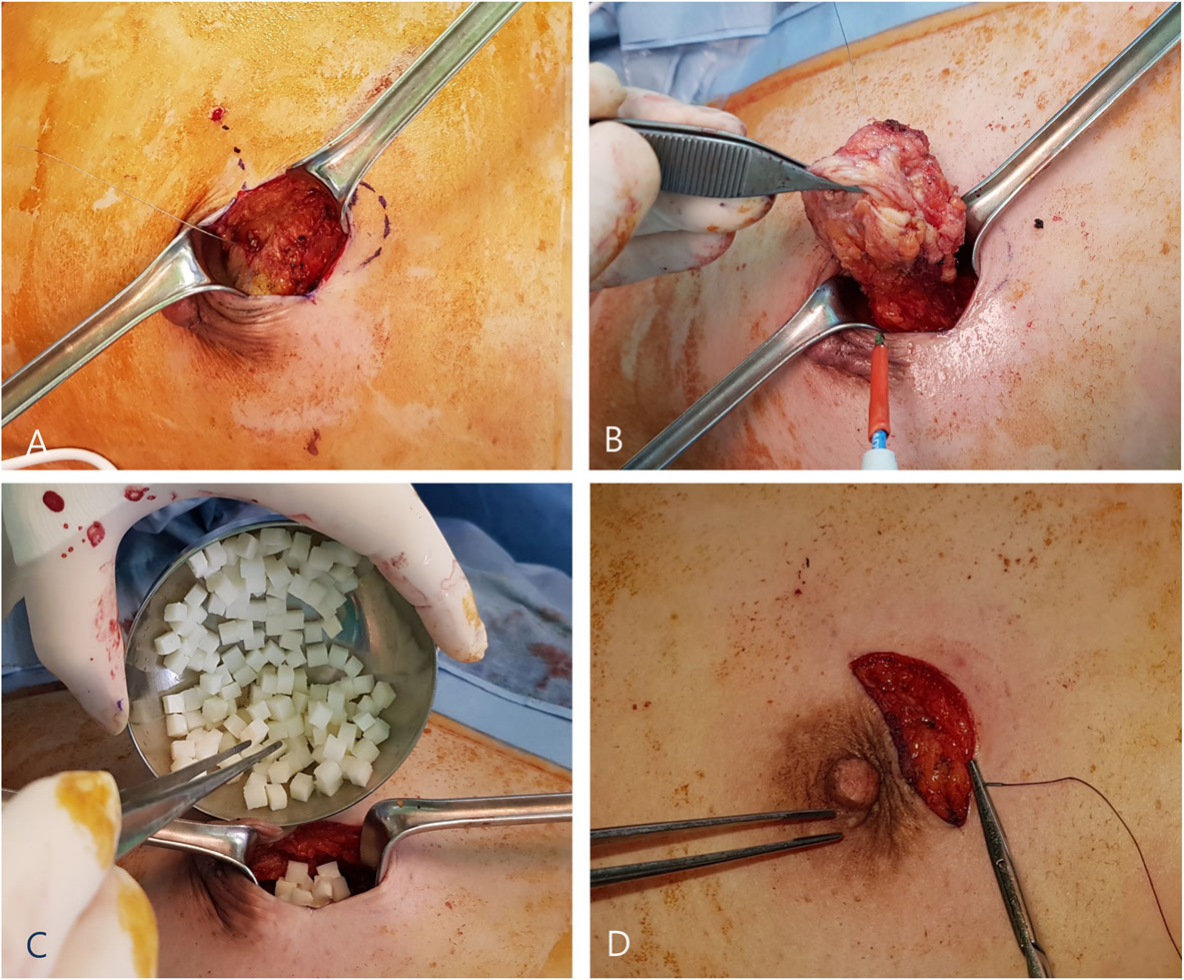
A subcutaneous fat flap was created after skin incision (**a**). Lumpectomy with wire localization was performed for the mass (**b**). The excision cavity was filled with soaked acellular dermal matrix pieces (**c**). The subcutaneous fat above the cavity was approximated to prevent abutting (**d**)

An intravenous second-generation cephalosporin (cefotetan) was used as the preoperative antibiotic. In patients with a history of allergy to cephalosporin, we used ciprofloxacin instead. A skin incision of 3–12 cm was made above the tumor mass, depending on the size. A 5–7-mm-thick subcutaneous fat flap was made, and the mass was excised enough to obtain a negative tumor margin. The excision cavity was then filled with the diced ADM pieces. We then covered the ADM-filled cavity by approximating the breast tissue and subcutaneous fat above it to prevent the ADM from abutting the skin directly (Fig. 3). The amount of ADM was chosen in such a way that it sufficiently filled the cavity but did not cause bulging of the breast. Only one pack of the product was used per person. If the defect was too large to be filled with diced ADM pieces alone, additional minimal tissue displacement was performed. All procedures were performed by three surgeons with over 7 years of experience in oncologic breast surgery.

### Survey of satisfaction and complications

The primary endpoint of the study was patient and surgeon satisfaction with the surgery outcomes 6 months postoperatively. Both cosmetic satisfaction and overall satisfaction were assessed on a 10-point scale using a questionnaire: scores of 1–4 were classified as dissatisfied; 5–6, neutral; 7–8, satisfied; and 9–10, strongly satisfied. The satisfaction of the patients and the surgeons was evaluated separately.

The secondary endpoint was postoperative complications. Complications were assessed 2 weeks and 6 months after surgery and were defined as the need for any kind of intervention.

Statistical analysis was performed using the paired samples *t* test to compare patient satisfaction with operator satisfaction. The chi-square test was used to compare the complication rate in subgroup analysis. A two-tailed *p* value less than 0.05 was considered statistically significant. Statistical analyses were performed using the R software (R Core Team [2013] R: A language and environment for statistical computing. R Foundation for Statistical Computing, Vienna, Austria. URL http://www.R-project.org/).

## Results

A total of 118 patients were included in the analysis. Their mean age was 51.7 years. The clinical characteristics of the patients are shown in Table 1. One patient was lost to follow-up 1 month after the operation, resulting in 117 patients completing the satisfaction questionnaire and complication evaluation after 6 months.

**Table 1:** Clinical characteristics of breast cancer patients (N=118) undergoing volume replacement with diced acellular dermal matrix

Characteristics	*N* (%)
Age (years)	
20–50	49 (44.9%)
> 50	69 (55.1%)
*T* stage	
Tis	17 (14.4%)
T1	63 (53.4%)
T2	14 (11.9%)
ypTis	1 (0.8%)
ypT0	4 (3.4%)
ypT1	7 (5.9%)
ypT2	9 (7.6%)
ypT3	3 (2.5%)
Pathology	
DCIS	17 (14.4%)
IDC	88 (74.6%)
ILC	2 (1.7%)
Others	11 (9.3%)
Location of cancer in breast	
Upper	88 (74.6%)
Central	10 (8.5%)
Lower	20 (16.9%)
Resection volume (cm^3^)	
0–50	62 (52.5%)
50–100	32 (27.1%)
> 100	24 (20.3%)

*DCIS* ductal carcinoma in situ, *IDC* invasive ductal carcinoma, *ILC* invasive lobular carcinoma

The mean satisfaction score for the cosmetic outcome on a 10-point scale was 9.7 (± 0.8) in the patient group and 9.7 (± 0.8) in the surgeon group (*p* = 0.250). The average score for overall satisfaction was 9.4 (± 1.0) in the patient group and 9.5 (± 1.1) in the surgeon group (*p* = 0.001). The responses in the cosmetic and overall satisfaction questionnaire revealed that more than 90% of the patients were strongly satisfied (Table 2).

**Table 2:** Satisfaction with cosmetic and overall surgical outcomes in patients (*N* = 117) using acellular dermal matrix

		Cosmetic outcome		Overall outcome	
Score		Surgeon *N*(%)	Patient *N*(%)	Surgeon *N*(%)	Patient *N*(%)
Strongly satisfied	10	91 (77.8)	92 (78.6)	78 (66.7)	83 (70.9)
	9	16 (13.7)	18 (15.4)	23 (19.7)	23 (19.7)
Satisfied	8	8 (6.8)	5 (4.3)	9 (7.7)	6 (5.1)
	7	1 (0.9)	0	3 (2.6)	0
Neutral	6	0	1 (0.9)	2 (1.7)	4 (3.4)
	5	0	1 (0.9)	1 (0.9)	1 (0.9)
Dissatisfied	4	1 (0.9)	0	1 (0.9)	0
	3	0	0	0	0
	2	0	0	0	0
	1	0	0	0	0
Average score		9.7	9.7	9.4	9.5

A total of 24 patients (20.5%) developed a complication until 6 months follow-up. Among the patients with a complication, 10 patients (8.5%) underwent reoperation. Two patients required removal of the ADM filling due to RBS and hematoma, respectively. The different complications with their rates after 2 weeks and 6 months are summarized in Tables 3 and 4, respectively.

**Table 3:** Postoperative complication rates in 118 breast cancer patients after volume replacement on postoperative day 14

	Incidence	Reoperation	ADM removal
Seroma	4 (3.4%)	0	0
Red breast syndrome	1 (0.8%)	0	0
Infection	0	0	0
Hematoma	6 (5.1%)	4 (3.4%)	0
Wound edge necrosis	1 (0.8%)	1 (0.8%)	0
Fat necrosis	1 (0.8%)	1 (0.8%)	0
Total	13 (11.0%)	6 (5.1%)	0

**Table 4:** Postoperative complication rates in 117 breast cancer patients after volume replacement 6 months postoperatively

	Incidence	Reoperation	ADM removal
Seroma	7 (6.0%)	0	0
Red breast syndrome	3 (2.5%)	1 (0.9%)	1 (0.9%)
Infection	3 (2.5%)	0	0
Hematoma	6 (5.1%)	4 (3.4%)*	1 (0.9%)
Wound edge necrosis	1 (0.9%)	1 (0.9%)	0
Fat necrosis	4 (3.4%)	4 (3.4%)	0
Total	24 (20.5%)	10 (8.5%)	2 (1.7%)

The most common postoperative complication was a seroma. Four patients developed a seroma within 2 weeks of surgery, and three patients developed a seroma after chemotherapy. The condition improved in all patients after aspiration. RBS occurred in three patients (2.5%): one case occurred within 2 weeks after surgery and two cases developed after radiotherapy (RT). The condition in two patients improved after receiving antihistamine treatment, but one patient who developed RBS after RT had to undergo ADM removal.

Subgroup analysis of complications was performed. The incidence of complications was higher when the primary tumor was located in the central part of the breast. The complication rate for tumors located in the upper part of the breast was 17/88 (19.3%), 5/10 (50.0%) for tumors located in the central part, and 2/20 (10.0%) for tumors located in the lower part of the breast (*p* = 0.033). The complication rate increased as the resection volume increased. The complication rate was 4/62 (6.5%) for a resection volume less than 50 cm^3^, 7/32 (21.9%) for resection volumes between 50 and 100 cm^3^, and 13/24 (54.2%) for resection volumes greater than 100 cm^3^ (*p* < 0.001). The rate of complications in the neoadjuvant chemotherapy group was significantly higher at 45.8% (11/24) than that in the non-neoadjuvant group (13.8% [13/94]) (*p* = 0.002). Among the 12 patients with a history of smoking and the 8 patients with diabetes, none had complications.

**Fig. 4:**
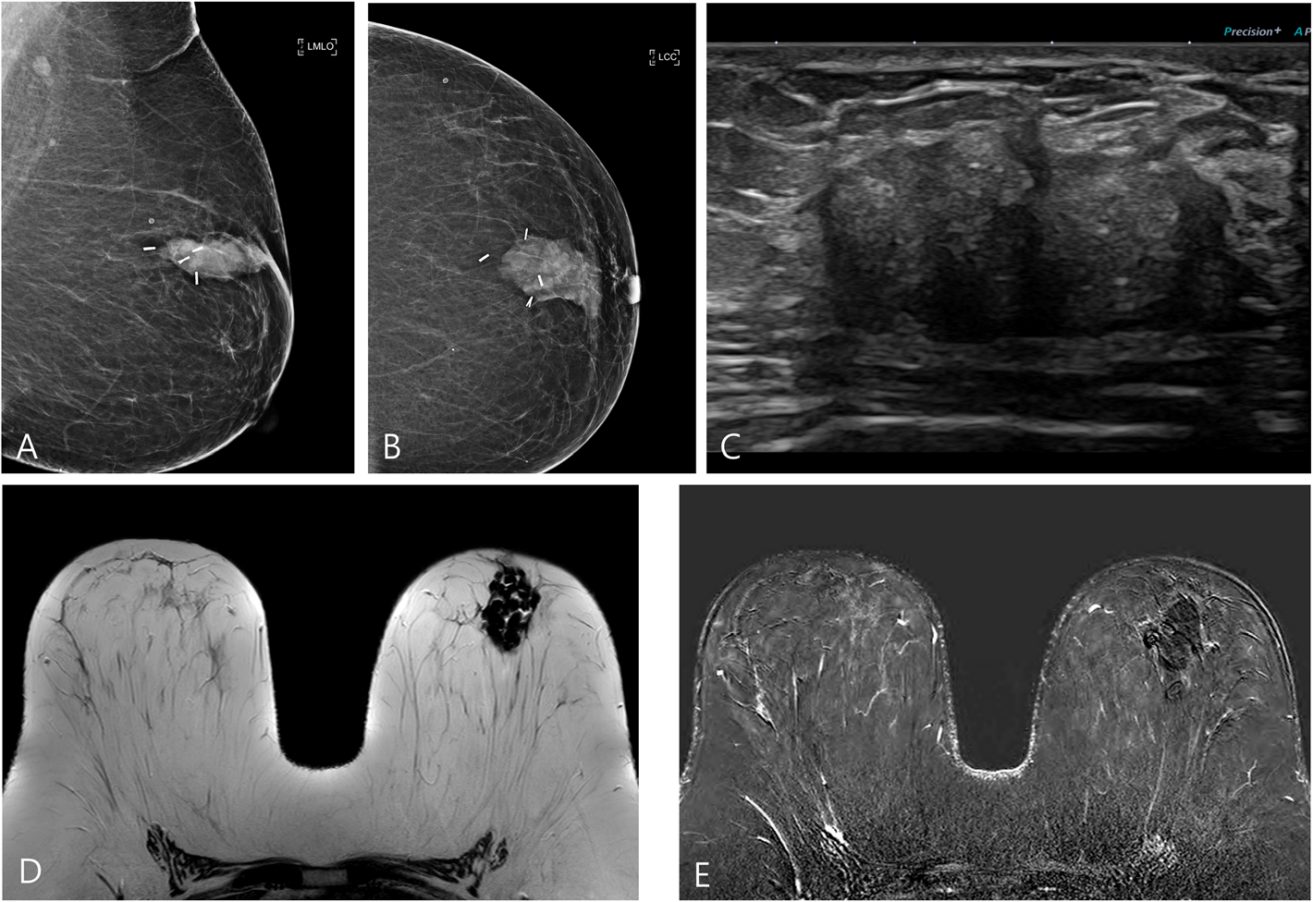
Six-month follow-up studies of a 42-year-old woman who underwent breast-conserving surgery with ADM. Mammography showed ADM as a mass with a well circumscribed margin on the left mediolateral oblique view (**a**) and left cranio-caudal view (**b**). The diced ADM was observed to the same echogenicity as fibroglandular parenchyma on B-mode ultrasonography (**c**). The ADM showed low signal intensity on T2-weighted imaging (**d**) and no enhancement on contrast-enhanced magnetic resonance imaging (**e**). ADM = acellular dermal matrix

One hundred seventeen patients underwent mammography, breast ultrasonography (USG), and magnetic resonance imaging (MRI) for follow-up at 6 months after surgery (Fig. 4). ADM was most clearly observed by USG. Postoperatively, on the follow-up sonogram, the gap with the matrix disappeared naturally as the seroma was absorbed. In all the patients, except for two patients in whom ADM was surgically removed, no morphological changes were observed in the ADMs using USG at the 6-month and 1-year follow-up. In one patient, a rim-enhancing mass was found around the ADM on contrast-enhanced MRI. The core needle biopsy results were lymphohistiocytic cell infiltration with eosinophils and fibrosis. This mass was not seen on the 1-year follow-up MRI.

## Discussion

ADM can be classified as human, porcine, or bovine ADM, depending on the source. The lowest complication incidence in breast cancer patients is associated with human ADM [11].

When using ADM, inflammation without infection is called RBS and has been reported in 1.7–6% of cases [11–13]. RBS is a delayed-type hypersensitivity reaction [14, 15]. In this study, its incidence was 2.5%, which was comparable to that in other reports.

We used a 10-point scale to evaluate the satisfaction level, including overall satisfaction and satisfaction with the shape. Unlike in other studies, the surgeons also participated in the survey, and their responses were compared with those of the patients. The average patient satisfaction score was 9.8 (± 0.7), and the overall satisfaction score was 9.6 (± 1.0). The surgeons’ cosmetic evaluation was not different from that of their patients. Overall satisfaction was slightly worse in the surgeon group. Complications, such as RBS, which are not seen in other surgeries without ADM, may have affected this result.

In 120 consecutive traditional BCSs that were performed before the new technique, complications such as seroma (10.8%), hematoma (2.5%), infection (5.8%), and fat necrosis (7.5%) occurred at 6 months of follow-up. The overall complication rate was 26.7% and reoperation was required in 7.5% of cases. Fat necrosis is a complication causing symptoms in the patient and sometimes requiring biopsy to differentiate it from malignancy. Fat necrosis occurs from 4.6 to 100%, depending on the surgical procedure [16, 17]. Our surgical method reported a lower fat necrosis rate (3.4%) than that of traditional BCS because it either did not make or minimized pedicled fat flap. Another advantage of ADM is that it is composed of cells that are resistant to radiation and have already been applied irradiation. Radiation therapy is also a risk factor of fat necrosis [18].

In this study, neoadjuvant chemotherapy, the central location of breast cancer, and high resection volume over 100 cm^3^ were risk factors for postoperative complications. However, we do not suggest selection criteria for this surgery. Seroma and hematoma accounted for 54.2% of all complications. Seroma with wound bulging or discharge improved after aspiration. Hematoma occurred in 6 patients: 5 cases occurred in the first 38 cases, and only 1 case occurred in the 80 subsequent cases. Four of these patients required reoperation. Therefore, it is necessary to consider the delicate hemostasis and prophylactic needle aspiration in patients with have associated risk factors.

One limitation of this study was that the neovascularization of ADM had not been confirmed histologically in our patients. After AMD implantation, neovascularization occurs between 1 and 6 months postoperatively [7]. During this period, most patients undergo chemo- and/or RT, which has already been shown to limit ADM remodeling [19]. In our study, 45.8% of all complications occurred between 2 weeks and 6 months.

## Conclusions

Immediate filling of defects with ADM was a safe and satisfactory BCS procedure in breast cancer patients. However, patients who underwent volume resections of greater than 100 cm^3^ and neoadjuvant chemotherapy had relatively higher complication rates. Evaluation of long-term cosmetic satisfaction as well as new approaches to reduce complications in high-risk groups is warranted.

## Data Availability

The datasets during and/or analyzed during the current study are available from the corresponding author on reasonable request.

## Abbreviations

ADM: Acellular dermal matrix
BCS: Breast-conserving surgery
MRI: Magnetic resonance imaging
RBS: Red breast syndrome
RT: Radiotherapy
USG: Ultrasonography
